# Facial Neuralgia Cured by a New Surgical Operation

**Published:** 1881-01

**Authors:** 


### FACIAL NEURALGIA CURED BY A NEW SURGICAL OPERATION.

The following case is from the *British Medical Journal*, by Dr. Aug. Brown. In April of this year, a lady, aged fifty-six, who had suffered many years from a most severe facial neuralgia, called upon me, and implored me to do something for her relief. I shall not readily forget the careworn expression of her face as she related to me the terrible nature of her sufferings. She told me that, for a period of upward of ten years she had endured the most fearful torture from constant attacks of neuralgia, which caused her to scream, and left her in an exhausted condition; and that, although she had incurred very considerable expense to obtain relief, she had failed to do so; and that the attacks were gradually increasing in violence, frequency and extent. She also informed me that she had been an in-patient, for some weeks, in the London Hospital, under the care of Dr. Fenwick, and that she had left that institution no better. I need not enumerate the various medicines and remedies which had been tried in this case — ice, electricity, etc. — for all alike had failed; even sub-cutaneous injections, although at first mitigating the paroxysms, began to lose their influence. Impressed by the supplications of my patient, I promised to do something for her. After considering the case for a week, I resolved upon a plan which I carried out on May 11th, 1880. In this case, the pain commenced in the mental nerve of the right side, just at its exit from the mental foramen; from this spot it ran backward to the front of the ear, then upward to the vertex, forward to the frontal nerve, down the right side of the face and neck to the arm, and backward to the scapula.

On examining the mouth, I found the gum, above the starting point of the pain, of a veined and congested appearance, thickened, and harder to the touch than the gum of the opposite side. The tongue was white and tremulous, all the teeth had been extracted. Six years ago she had a portion of the alveolar process removed; the idea then being that the pain was produced by the pressure of a buried stump of a tooth; but the operation proved that this was not the case. Mr. Penny and Dr. Rowntree kindly assisted me with the operation. As soon as the chloroform took effect, I made an incision along the lower border of the jaw, and dissected up a flap till I reached the mental foramen. I then ran into the foramen a red-hot steel wire for a quarter of an inch or so, and throughly destroyed the nerve. On withdrawing the wire, the artery bled considerably, and I was obliged to plug the foramen. This plug was the cause of some amount of suppuration and delay in the healing of the wound. However, it came away in a few days in the discharges, and then the wound healed kindly, and my patient, from that time, has been entirely free from pain, and is now restored to health. Anything more satisfactory than the result of this operation I have never known. She is now able to take food without fear, to sleep without narcotics, her tongue has regained its color, and she now takes an interest in her household affairs. Much, lately, has been said and written about nerve-stretching; but the result of this operation proves that in the cautery we have another remedy upon which we may depend, and which, in many instances, may supersede nerve-stretching; also one which possibly may be of great benefit in tetanus.

